# Association between long working hours and serum gamma-glutamyltransferase levels in female workers: data from the fifth Korean National Health and Nutrition Examination Survey (2010-2011)

**DOI:** 10.1186/s40557-014-0040-1

**Published:** 2014-12-01

**Authors:** Seung-Gwon Park, Yong-Jin Lee, Jung-Oh Ham, Eun-Chul Jang, Seong-Woo Kim, Hyun Park

**Affiliations:** Department of Occupational & Environmental Medicine, Soonchunhyang University Cheonan Hospital, 31, Soonchunhyang 6-gil, Dongnam-gu, Cheonan-si, Chungcheongnam-do, 330-930 Republic of Korea

**Keywords:** Long working hours, Gamma-glutamyltransferase, Cardiovascular disease

## Abstract

**Objectives:**

The present study investigated the association between long working hours and serum gamma-glutamyltransferase (GGT) levels, a factor influencing the incidence of cardiovascular disease.

**Methods:**

Data from the fifth Korean National Health and Nutrition Examination Survey (2010–2011) were used to analyze 1,809 women. Subjects were divided into three groups based on the number of weekly working hours: ≤29, 30–51, and ≥52 hours per week. Complex samples logistic regression was performed after adjusting for general and occupational factors to determine the association between long working hours and high serum GGT levels.

**Results:**

The prevalence of high serum GGT levels in groups with ≤29, 30–51, and ≥52 working hours per week was 22.0%, 16.9%, and 26.6%, respectively. Even after adjusting for general and occupational factors, those working 30–51 hours per week had the lowest prevalence of high serum GGT levels. Compared to those working 30–51 hours per week, the odds ratios (OR) of having high serum GGT levels in the groups with ≥52 and ≤29 working hours per week were 1.56 (95% confidence interval [CI], 1.10–2.23) and 1.53 (95% CI, 1.05–2.24), respectively.

**Conclusions:**

Long working hours were significantly associated with high serum GGT levels in Korean women.

## Introduction

Long working hours lead to poor health habits such as smoking and alcohol consumption as well as sleep deprivation and excessive stress. In turn, these poor habits adversely affect different aspects of health, including the cardiovascular, gastrointestinal, and immune systems as well as mental health [1-3]. Among several negative health consequences, cardiovascular disease and its risk factors are the primary focus of many studies. Thus far, studies on the risk factors of cardiovascular diseases indicate long working hours are associated with increased incidence rates of high blood pressure, diabetes, acute myocardial infarction, and coronary artery disease [4-8].

Caruso et al. [3] reported that two reasons for long working hours include a workers’ desire to increase their earnings as well as a tendency of employers to keep costs low by avoiding hiring additional employees. In addition, Artazcoz et al. [9] suggested that the stresses of being a breadwinner and economic vulnerability are important causes of long working hours. Because the overtime pay rate is high in the Republic of Korea (hereafter Korea), Korean workers have a strong preference for working overtime. Moreover, the lack of flexibility in hiring practices as well as good fringe benefits collectively discourages employers’ from hiring additional employees. As such, the culture of long working hours is much more prevalent in Korea than other countries [10]. Furthermore, female workers in Korea generally shoulder the bulk of domestic work in their families. In fact, female workers in Korea spend 3–6 times longer on domestic work than their male counterparts do [11]; moreover, the duration of domestic work performed by Korean men is the shortest among all Organization for Economic Co-operation and Development (OECD) countries [12]. Despite the high prevalence of long working hours among women, most studies on long working hours have exclusively focused on men [13].

Gamma-glutamyltransferase (GGT) has been used as a biomarker of alcohol intake and hepatocellular damage [14]. However, recent reports suggest GGT is also a biomarker of oxidative stress [15]. Oxidative stress is involved in the pathophysiology of cardiovascular disease; therefore, serum GGT has been receiving increasing attention as a potential early indicator of cardiovascular disease [16-18]. We investigated the relationship between long working hours and serum GGT levels in Korean women.

## Materials and Methods

### Subjects

The Korean National Health and Nutrition Examination Survey (KNHANES) uses a rolling survey sampling wherein the samples of each year serve as an independent probability sampling pool and the samples sharing similar characteristics in each sample year are selected. To collect a representative Korean population, a stratified multistage-clustered probability design is used to sample enumeration districts. The enumeration districts were created as follows. First, the cities and provinces of Korea were stratified. Then, the general housing area was stratified into 26 levels with respect to sex, age group, and population ratio. Apartment complex areas were stratified into 24 levels according to the price per square meter and average number of square meters per apartment. After residences were subjected to this 2-stage stratification, 20 residence units were extracted per enumeration district; thus, a total of 3,840 residences were extracted from 192 enumeration districts.

The KNHANES comprises health, clinical examination, and nutritional surveys. While health and nutrition surveys were conducted by trained investigators, the health behavior aspects of the health survey including smoking status and alcohol consumption were assessed using self-reported data.

Data from the fifth KNHANES (2010–2011) were used because serum GGT levels were only collected during this period. Among the study population of 21,527 people, this study involved 16,528 participants (participation rate, 76.7%). Among 1,905 pre-menopausal women between 20 and 49 years old who were employed, 54 women with hepatitis B or C, or liver cirrhosis as well as 42 with alanine aminotransferase levels ≥36 IU/L were excluded to avoid the confounding effect of liver disease. Thus, a total of 1,809 women were analyzed.

### Measures

In order to measure serum GGT (dependent variable), an enzymatic activity assay (G5CMP) was performed using the Hitachi Automatic Analyzer 7600 (Hitachi, Japan). Participants with serum GGT levels ≤20 and ≥21 IU/L were classified as the normal and high serum GGT groups, respectively [19]. Working hours per week (independent variable) was classified into three groups: ≤29 hours (defined as part-time employment by the OECD) [20], 30–51 hours, and ≥52 hours (defined as long working hours by the Labor Standards Act in Korea) [21].

Age was categorized between 20–39 or 40–49 years. Body mass index (BMI) was categorized as <18.5 (underweight), 18.5–22.9 (normal), or ≥23 (overweight) kg/m^2^. The volume and frequency of alcohol consumption were asked using closed-ended questions; therefore, to calculate weekly alcohol consumption, the mean volume and frequency of alcohol consumption were multiplied. The products were categorized as ≤2.5 or >2.5 standard-sized drinks per week. In addition, participants were categorized as non-smokers, ex-smokers, or current smokers. The number of hours sleep per day was categorized as ≤5, 6–8, or ≥9 hours. Walking at least 30 minutes per day 5 days per week was considered regular walking. The frequency of coffee consumption was categorized as <1 or ≥1 cup of coffee per day. Work performed between 6:00 am to 6:00 pm was categorized as day work, and work performed outside this time was categorized as non-day work. Moreover, participants were classified as salary workers, being self-employed, or unpaid family workers. Manual work included agricultural forestry and fishery work, crafts and related trades, equipment, machine operation and assembly, and elementary work. All other occupations were categorized as non-manual work.

### Statistical analysis

Because the KNHANES uses a complex sample design, a survey module for complex samples and survey weights were applied. To analyze the characteristics of the study population and factors influencing serum GGT levels, a complex samples *χ*^2^ tests were performed to calculate estimated percentages. In addition, complex samples logistic regression was performed to calculate odds ratios (OR) and 95% confidence intervals (CI) of high serum GGT levels after adjusting for variables significantly associated with GGT levels in the univariate analysis and work type. All statistical analyses were performed using SPSS version 18.0 (SPSS Inc., Chicago, IL, USA), and the level of significance was set at *p* <0.05.

## Results

### General and occupational characteristics

The distribution of working hours per week included 400 (21.0%), 1,054 (57.8%), and 355 (21.2%) women who worked ≤29, 30–51, and ≥52 hours per week, respectively. Those aged 40–49 tended to be the most likely to work ≥52 hours per week (41.4%). BMI did not differ significantly among groups. The proportions of women with >2.5 alcoholic drinks per week (37.9%), currently smoking (13.7%), and sleeping <5 hours of per night (9.4%) were higher among those working ≥52 hours per week than that of other groups. Non-day workers was in the ≤29 hour group (32.9%), and salary workers were commonly worked 30–51 hours per week (81.9%). There were no significant differences between occupations and working hours per week (Table 1).

**Table 1:** **General and occupational characteristics**

**Variables**	**Total**	**Weekly working hours**			***P*** **-value** ^**†**^
		**≤29 N (%** * **)**	**30–51 N (%** * **)**	**≥52 N (%** * **)**	
Total	1,809 (100.0)	400 (21.0)	1,054 (57.8)	355 (21.2)	
Age (years)					
20-39	1,150 (65.0)	256 (64.8)	691 (67.5)	203 (58.6)	0.039
40-49	659 (35.0)	144 (35.2)	363 (32.5)	152 (41.4)	
BMI (kg/m^2^)					
Underweight (<18.5)	182 (10.8)	38 (10.3)	120 (12.2)	24 (7.4)	0.172
Normal (18.5–22.9)	1,307 (71.1)	290 (70.6)	765 (71.1)	252 (71.4)	
Overweight (≥23.0)	317 (18.1)	70 (19.1)	169 (16.7)	78 (21.2)	
Alcohol consumption (glasses/week)					
≤2.5	1,217 (66.3)	291 (72.5)	708 (65.5)	218 (62.1)	0.035
>2.5	592 (33.7)	109 (27.5)	346 (34.5)	137 (37.9)	
Smoking status					
Non-smoker	1,532 (82.3)	343 (86.1)	904 (82.4)	285 (78.3)	0.040
Ex-smoker	136 (8.5)	30 (8.0)	76 (8.8)	30 (8.0)	
Current smoker	141 (9.2)	27 (5.8)	74 (8.8)	40 (13.7)	
Sleep duration (hours/day)					
≤5	151 (9.0)	30 (8.7)	85 (9.0)	36 (9.4)	0.028
6–8	1,527 (83.5)	327 (79.4)	897 (83.9)	303 (86.5)	
≥9	131 (7.5)	43 (11.9)	72 (7.2)	16 (4.1)	
Regular walking					
Yes	658 (37.3)	152 (40.6)	388 (37.9)	118 (32.5)	0.135
No	1,150 (62.7)	248 (59.4)	665 (62.1)	237 (67.5)	
Coffee intake (cups/day)					
<1	504 (37.8)	87 (31.2)	304 (39.2)	113 (40.2)	0.130
≥1	840 (62.2)	200 (68.8)	479 (60.8)	161 (59.8)	
Working type					
Day work	1,478 (81.6)	281 (67.1)	907 (85.6)	290 (85.0)	<0.001
Non-day work	317 (18.4)	117 (32.9)	141 (14.4)	59 (15.0)	
Occupation					
Manual work	266 (15.6)	61 (16.4)	145 (14.8)	60 (17.1)	0.633
Non-manual work	1,542 (84.4)	339 (83.6)	909 (85.2)	294 (82.9)	
Employment status					
Salary worker	1,127 (73.8)	206 (64.7)	746 (81.9)	175 (60.8)	<0.001
Self-employed	289 (19.1)	87 (26.1)	108 (13.1)	94 (28.3)	
Unpaid family worker	107 (7.2)	26 (9.2)	42 (5.0)	39 (10.9)	

*Estimate percentage.

^†^ *χ* ^2^ test.

### General characteristics and high serum GGT levels

The prevalence of high serum GGT levels in women aged 20–39 and 40–49 years was 16.6% and 26.4%, respectively. Moreover, the prevalence of high serum GGT levels in underweight, normal, and overweight subjects was 11.2%, 15.5%, and 42.5%, respectively. For alcohol consumption, high serum GGT levels was significantly more prevalent among subjects who consumed >2.5 drinks per week (26.4%) than those who consumed ≤2.5 drinks per week (16.8%). In addition, a high serum GGT level was more prevalent in current smokers (40.4%) than ex-smokers (18.4%) and non-smokers (17.9%). Women who did not walk regularly had higher serum GGT levels than those who walked regularly did (22.0% vs. 16.8%). However, high serum GGT was not significantly different across sleep duration or caffeine intake groups (Table 2).

**Table 2:** **Prevalence of high serum gamma-glutamyltransferase levels across general characteristics**

**Variable**	**Serum GGT** *		***P*** **-value** ^**§**^
	**Normal level group**	**High level group** ^**†**^	
	**N (%** ^**‡**^ **)**	**N (%** ^**‡**^ **)**	
Total	1,470 (80.0)	339 (20.0)	
Age (years)			
20-39	974 (83.4)	176 (16.6)	<0.001
40-49	496 (73.6)	163 (26.4)	
BMI (kg/m^2^)			
Underweight (<18.5)	158 (88.8)	24 (11.2)	<0.001
Normal (18.5–22.9)	1,113 (84.5)	194 (15.5)	
Overweight (≥23.0)	197 (57.5)	120 (42.5)	
Alcohol consumption (glasses/week)			
≤2.5	1,029 (83.2)	188 (16.8)	<0.001
>2.5	441 (73.6)	151 (26.4)	
Smoking status			
Non-smoker	1,271 (82.1)	261 (17.9)	<0.001
Ex-smoker	112 (81.6)	24 (18.4)	
Current smoker	87 (59.6)	54 (40.4)	
Sleep duration (hours/day)			
≤5	114 (74.3)	37 (25.7)	0.249
6–8	1,251 (80.6)	276 (19.4)	
≥9	105 (79.5)	26 (20.5)	
Regular walking			
Yes	553 (83.2)	105 (16.8)	0.030
No	916 (78.0)	234 (22.0)	
Coffee intake (cups/day)			
<1	405 (79.3)	99 (20.7)	0.471
≥1	687 (81.3)	153 (18.7)	

*Gamma-glutamyltransferase.

^†^Serum GGT ≥21 IU/L.

^‡^Estimated percentage.

^§^ *χ* ^2^ test.

### Occupational characteristics and high serum GGT levels

The prevalence of high serum GGT levels was the lowest among subjects working 30–51 hours per week (16.9%) followed by those working ≤29 hours per week (22.0%) and ≥52 hours per week (26.6%). In addition, salary workers exhibited the lowest prevalence of high serum GGT levels (16.8%). However, no significant differences across occupation or working type groups and serum GGT levels were found (Table 3).

**Table 3:** **Prevalence of high serum gamma-glutamyltransferase levels across occupational characteristics**

**Variable**	**Serum GGT** *		***p*** **-value** ^**§**^
	**Normal level group**	**High level group** ^**†**^	
	**N (%** ^**‡**^ **)**	**N (%** ^**‡**^ **)**	
Weekly working hours			
≤29	321 (78.0)	79 (22.0)	0.002
30–51	882 (83.1)	172 (16.9)	
≥52	267 (73.4)	88 (26.6)	
Working type			
Day work	1,200 (79.9)	278 (20.1)	0.820
Non-day work	261 (80.6)	56 (19.4)	
Occupation			
Manual work	212 (78.2)	54 (21.8)	0.489
Non-manual work	1,257 (80.3)	285 (19.7)	
Employment status			
Salary worker	947 (83.2)	180 (16.8)	<0.001
Self-employed	218 (71.6)	71 (28.4)	
Unpaid family worker	75 (70.0)	32 (30.0)	

^*^Gamma-glutamyltransferase.

^†^Serum GGT ≥21 IU/L.

^‡^Estimated percentage.

^§^ *χ* ^2^ test.

### Working hours and high serum GGT levels

Compared to those working 30–51 hours per week, the OR of having high serum GGT levels among those working ≤29 and ≥52 hours per week were 1.39 (95% CI, 0.99–1.94) and 1.78 (95% CI, 1.30–2.44), respectively. Model 1, adjusted for general characteristics (age, BMI, alcohol consumption, smoking status, and regular walking), the OR of having high serum GGT levels among those working ≤29 and ≥52 working hours per week were 1.51 (95% CI, 1.03–2.19) and 1.52 (95% CI, 1.07–2.15) respectively. Model 2, adjusted for the variables from model 1 as well as work type and employment status, the OR were 1.53 (95% CI, 1.05–2.24) and 1.56 (95% CI, 1.10–2.23) among those working ≤29 and ≥52 working hours per week, respectively (Table 4).

**Table 4:** **Associations between weekly working hours and high serum gamma-glutamyltransferase levels**

**Working hours/week**	**Crude**		**Model 1** ^*^		**Model 2** ^**†**^	
	**OR** ^**‡**^	**95%** **CI** ^**§**^	**OR** ^**‡**^	**95%** **CI** ^**§**^	**OR** ^**‡**^	**95%** **CI** ^**§**^
≤29	1.39	0.99–1.94	1.51	1.03–2.19	1.53	1.05–2.24
30–51	1		1		1	
≥52	1.78	1.30–2.44	1.52	1.07–2.15	1.56	1.10–2.23

*Adjusted for general characteristics (i.e., age, BMI, alcohol consumption, smoking status and regular walking).

^†^Adjusted for general and occupational characteristics (i.e., Model 1 + working type, and employment status).

^‡^Odds ratio, ^§^95% confidence interval.

## Discussion

Among Korean female workers, the prevalence of high serum GGT levels was higher among those working long hours (≥52 hours per week) than those working 30–51 hours per week even in the fully adjusted model.

The definition of long working hours varies by country and institution. The Labor Standards Act in Korea has set the weekly working hours at <40 hours and <52 hours including overtime [21]. However, the International Labor Organization and the European Union define 48 hours as appropriate number of weekly working hours [22,23]. According to the 2012 survey in Korea on labor conditions by employment type, the average monthly working hours was 173.7 [24]. One report from the OECD indicates the annual working hours of Koreans has decreased steadily from a peak of 2,512 hours in 2000; however, Koreans still have the longest annual working hours among all OECD countries, with 2,193 hours in 2010 [25].

Many recent studies have investigated the influence of long weekly working hours on cardiovascular health. Hayashi et al. [5] reported that 24-hour ambulatory blood pressure is higher among office workers with long weekly working hours than among office workers in their control group. Iwasaki et al. [4] reported that certain age groups have increased systolic blood pressure because of increased fatigue due to long weekly working hours. In addition, Kawakami et al. [6] found that working >50 hours per week increases the risk of contracting non-insulin-dependent diabetes by 3.7 fold, and a meta-analysis by Virtanen et al. [8] revealed that long weekly working hours increases the risk of coronary artery disease by approximately 1.4 fold.

Long working hours can affect an individual’s health in several ways. By working long hours, an individual can experience physiological changes in blood pressure, hormone excretion, and sympathetic nervous system activity among others. Furthermore, long working hours can result in negative lifestyle behaviors such as alcohol consumption, smoking, an unhealthy diet, and a lack of exercise. Thus, the combination of negative physiological and lifestyle changes can collectively increase one’s risk of cardiovascular disease [1]. The present study also revealed high prevalences of smoking, drinking, and inadequate sleep duration in those working ≥52 hours per week. Shields et al. [26] reported that increased working hours are associated with increased alcohol consumption in women. In addition, Steptoe et al. [27] reported that women who work long hours tend to smoke more cigarettes. Virtanen et al. [28] found that people working >55 hours per week not only slept fewer hours per day, but also experience difficulty falling asleep.

Serum GGT activity is elevated when glutathione, an antioxidant, exerts its cytoprotective effect by inhibiting free radicals produced by oxidative stress [14]. Increasing evidence indicating that serum GGT is a biomarker of oxidative stress [15] consequently revealed that serum GGT is associated with diseases including high blood pressure, diabetes, metabolic syndrome, stroke, and coronary artery disease. Moreover, serum GGT may explains their respective pathogenesis [16-18,29]. Interest in serum GGT has been further amplified because of its association with other early predictors of cardiovascular disease, including high-sensitivity C-reactive protein, pulse wave velocity, and the Framingham risk score [30,31].

In addition to conventional well-known factors (age, sex, and alcohol consumption), obesity, smoking, and physical activity also influence serum GGT levels. The present study corroborates obesity as a factor influencing GGT levels, because the overweight group exhibited the highest prevalence of high serum GGT levels. Furthermore, current smokers exhibited a higher prevalence of high serum GGT levels than ex- and non-smokers did. The prevalence of high serum GGT levels was significantly lower in regular walkers than in non-regular walkers, which is similar to the results of the study by Nilssen et al. [32]. Although previous studies report that coffee consumption is inversely associated with serum GGT levels [32,33], the present study found no significant association. The mechanism by which coffee reduces serum GGT levels is not fully understood; Nilssen et al. [32] also claim that different types of coffee (e.g., boiled, filtered, and instant) can have different effects on serum GGT levels.

In the present study, we found no significant difference in the prevalence of high serum GGT levels between day and non-day workers; this finding is concordant with those of Higashikawa et al. [34] and Knutsson et al. [35]. However, 70.0% of women who performed non-day work performed evening work, while only 30.0% performed night work or regular shift work. Therefore, the classical health effects of atypical work hours may not be applicable to the present study population.

In the present study, women who worked ≤29 hours per week were more likely to have high serum GGT levels than those who worked 30–51 hours did even after full adjustment. In their case–control study of 195 myocardial infarction patients, Sokejima et al. [10] found that patients who worked <7 and >11 hours per day had a higher risk of myocardial infarction than those who worked 7–9 hours per day did; thus, a U-shaped association between the number of daily working hours and risk of myocardial infarction was found. Morris et al. [36] also reported that decreased working hours due to unemployment could increase the risk of acute myocardial infarction. In addition, previously unhealthy individuals may have been overrepresented in the ≤29 hours per week work group in the present study. Therefore, the relationship between low working hours and health should be a target of future prospective studies.

The present study has some limitations. First, this was a cross-sectional study, so a causal relationship between long working hours and high serum GGT levels cannot be suggested. Furthermore, although the effects of long working hours on serum GGT levels are understood to be chronic in nature, the present study could not determine an appropriate working period for studying the chronic effects of long working hours. Second, because there is no precise definition or diagnostic standard for high serum GGT levels, the cut-off of high serum GGT levels used in the present study (≥21 IU/L) may not be a clinically accepted value. Thus, a different cut-off value may change our study findings. Lee et al. [15] reported a strong dose–response relationship of GGT levels within normal limits with oxidative stress and cardiovascular risk. However, to the best of our knowledge, there are no reports of a dose–response relationship between abnormal GGT levels and cardiovascular risk. Consequently, we used a relatively low cut-off to define high serum GGT to include both those with clinically abnormal levels and those with high GGT levels within reference range. In addition, because a comparison of the geometric means of serum GGT levels across the three groups showed results similar to those in this study, a different cut-off should not substantially alter the structure or results of the present study. Third, the present study targeted only women. Some studies have suggested that cardiovascular responses to stress differ between men and women [37]. Furthermore, the manifestations of stress-related behavioral changes may also differ between sexes [38]. Therefore, caution should be taken when applying the results of this study to men. Most conventional studies of long working hours and health have exclusively targeted men, and the few studies targeting women and their working hours involved only specific occupations. Therefore, the present study is meaningful because representative data on Korean women and a commonly used blood test parameter, serum GGT, were used to analyze the associations between long working hours and employee’s health.

## Conclusion

In conclusion, our study results suggest that long working hours are associated with high serum GGT levels in Korean women. Considering the results of many previous studies reporting an association between cardiovascular risk and serum GGT levels, serum GGT may be a useful biomarker for determining which employees require additional attention to their health. Thus, our study results have important implications in public health. Therefore, future, well-designed prospective studies addressing the aforementioned limitations of this study and those of previous studies are required to understand the relationship between these factors.
